# Comparison of the time to detection (TTD) of different blood culture systems and antibiotic adsorption capacity of different pediatric blood culture bottles of BD and bioMérieux

**DOI:** 10.1128/spectrum.02186-25

**Published:** 2025-11-21

**Authors:** Qing Meng, Xiaoya Liu, Wujiao Li, Yuhui Wu, Xiuhui Huang, Liangliang Kang, Zhihao Xing, Xuejuan Li, Lintao Zhou, Yunsheng Chen, Xiaoying Fu

**Affiliations:** 1 Department of Clinical Microbiology Laboratory, Shenzhen Children's Hospital, Shenzhen, Guangdong, China; 2 Department of Pharmacy, Shenzhen Children's Hospital, Shenzhen, Guangdong, China; 3 Department of Intensive Care Unit, Shenzhen Children's Hospital, Shenzhen, Guangdong, China; 4 Department of Pediatrics, The First Hospital of Lanzhou University, Lanzhou, Gansu, China; MultiCare Health System, Tacoma, Washington, USA

**Keywords:** blood culture, antimicrobial drugs, bloodstream infection, bacterial infection

## Abstract

We evaluated the time to detection (TTD) of pediatric blood culture bottles (BacT/Alert-PF, BacT/Alert-PF plus, and Bactec-PF) and instruments (BacT/Alert 3D, BacT/Alert VIRTUO, and Bactec FX) via *in vitro* simulations of bacterial and fungal bloodstream infections. We also assessed the antibiotic adsorption capacity of the three pediatric bottles under simulated peak antibiotic concentrations. Among all tested bacteria, the median TTDs for BacT/Alert VIRTUO with BacT/Alert-PF plus and for Bactec FX with Bactec-PF were 11.62 h and 13.48 h, respectively, both of which are shorter than those of BacT/Alert 3D with BacT/Alert-PF (17.25 h). Within the BacT/Alert 3D system, BacT/Alert-PF plus demonstrated a shorter median TTD than BacT/Alert-PF (13.92 h vs 17.25 h). Under identical bottle conditions (BacT/Alert-PF plus), BacT/Alert VIRTUO achieved a shorter median TTD than BacT/Alert 3D (11.62 h vs 13.92 h). In the antibiotic adsorption evaluation, Bactec-PF (51.3%) and BacT/Alert-PF plus (46.1%) achieved significantly higher detection rates than BacT/Alert-PF (13%) for ampicillin, oxacillin, ceftriaxone, ceftazidime, cefepime, cefoperazone/sulbactam, piperacillin/tazobactam, meropenem, amikacin, and vancomycin. The detection rates of Bactec-PF and BacT/Alert-PF plus varied by antibiotic–strain combination and increased with the level of bacterial resistance. All three bottles achieved 100% detection in the amikacin group, whereas the detection rates were 0% in the ceftriaxone and meropenem groups. These findings provide important guidance for selecting blood culture systems in patients who have received prior antibiotic therapy and for improving the prognosis of bloodstream infections.

**IMPORTANCE:**

Rapid reporting of positive blood cultures can directly influence physicians’ treatment decisions, thereby reducing mortality and improving patient outcomes. This study compared the time to detection (TTD) and antibiotic adsorption capacity of commonly used pediatric blood culture bottles and instruments, which are currently less evaluated. Providing optimized testing options for blood culture is essential to shorten the TTD and improve culture positivity rates in patients who have already received antibiotics.

## INTRODUCTION

Blood culture remains the gold standard for diagnosing bloodstream infections (BSIs), as it allows for the direct recovery and identification of causative pathogens. However, traditional blood culture has notable limitations, including a prolonged time to detection (TTD) and relatively low sensitivity, particularly in pediatric patients where the sample volume is limited. To overcome these challenges, rapid diagnostic technologies, as well as improvements in both blood culture bottle formulations and automated detection systems, have been developed in recent years with the aim of shortening the TTD and increasing diagnostic yield (1, 2).

Currently, two major automated blood culture systems are widely used worldwide: the Bactec FX system (BD Diagnostics, USA) and the BacT/Alert system (bioMérieux, France). Both systems employ continuous monitoring of CO₂ production to detect microbial growth; however, they differ in their incubation algorithms, optical sensors, and bottle formulations, which can influence their performance (3, 4). The more recent BacT/Alert VIRTUO system has further improved workflow efficiency by fully automating the loading process and reducing delayed incubation while also demonstrating earlier detection of positive cultures than the BacT/Alert 3D system (3).

Blood culture bottles themselves are equally important, as medium composition, resin or charcoal additives, and antibiotic neutralization capacity directly affect bacterial recovery. For adults, the BacT/Alert-Fastidious Antimicrobial Neutralization Aerobic (FA) plus bottle has been shown to shorten the TTD compared with the BacT/Alert-FA bottle and to improve the adsorption of certain antibiotics (5, 6). Pediatric formulations—including the Bactec-Pediatric Resin Medium (PF) aerobic bottle, the BacT/Alert-Pediatric Fastidious Antimicrobial Neutralization (PF) bottle, and its updated version, the BacT/Alert-PF plus bottle—are specifically designed for small inoculum volumes and are also occasionally used in adults (7). In 2018, bioMérieux modified the PF plus formulation (8), but published evaluations of its TTD performance and antibiotic adsorption capacity remain limited (9, 10).

Given these developments, systematic evaluation of pediatric blood culture bottles across different automated detection systems is necessary to guide clinical laboratory selection and optimize diagnostic yield in children. The present study compared four test combinations: (i) Bactec FX with Bactec-PF bottles, (ii) BacT/Alert 3D with BacT/Alert-PF bottles, (iii) BacT/Alert 3D with BacT/Alert-PF plus bottles, and (iv) BacT/Alert VIRTUO with BacT/Alert-PF plus bottles. We assessed the TTD of common pediatric pathogens under these conditions and further evaluated the recovery of American Type Culture Collection (ATCC) strains via Bactec-PF, BacT/Alert-PF, and BacT/Alert-PF plus bottles *in vitro* under simulated peak antibiotic concentrations, thereby systematically assessing bottle performance in the presence of antimicrobial agents.

## MATERIALS AND METHODS

### Materials

#### Strain collection

For TTD evaluation, clinical isolates were collected from pediatric patients (<18 years old) (11) at Shenzhen Children’s Hospital between January 2022 and March 2023. Duplicate isolates from the same patient were excluded, resulting in a total of 183 strains. These strains were obtained from various specimen types, including sputum (*n* = 52), blood (*n* = 41), urine (*n* = 38), stool (*n* = 21), pus (*n* = 12), sterile body fluids (*n* = 8), and secretions (*n* = 11). The species distributions are summarized in Table 3.

For antibiotic adsorption evaluation, ATCC reference strains with minimum inhibitory concentrations (MICs) or inhibition zone diameters within the susceptible range for the corresponding antibiotics were selected. These strains included *Escherichia coli* ATCC 25922, *Enterococcus faecalis* ATCC 29212, *Staphylococcus aureus* ATCC 29213, *Pseudomonas aeruginosa* ATCC 27853, *Streptococcus pneumoniae* ATCC 49619, and *Klebsiella pneumoniae* ATCC 700603. Detailed information is provided in Table 1.

**TABLE 1:** Antibiotic adsorption evaluation test: antibiotics and corresponding ATCC strains

Antibiotic	*Enterococcus faecalis* ATCC 29212		*Escherichia coli* ATCC 25922		*Staphylococcus aureus* ATCC 29213		*Streptococcus pneumoniae* ATCC 49619		*Pseudomonas aeruginosa* ATCC 27853		*Klebsiella pneumoniae* ATCC 700603	
	MIC range	Antimicrobial susceptibility	MIC range/inhibition zone diameter	Antimicrobial susceptibility	MIC range	Antimicrobial susceptibility	MIC range	Antimicrobial susceptibility	MIC range/inhibition zone diameter	Antimicrobial susceptibility	MIC range	Antimicrobial susceptibility
Ampicillin	0.5–2 mg/L	S	2–8 mg/L	S								
Oxacillin					0.12–0.5 mg/L	S						
Ceftriaxone			0.03–0.12 mg/L	S			0.03–0.12 mg/L	S				
Ceftazidime			0.06–0.5 mg/L	S					1–4 mg/L	S		
Cefepime			0.016–0.12 mg/L	S					0.5–4 mg/L	S	0.5–2 mg/L	S
Cefoperazone/sulbactam			27–33 mm^*a*^	S^*a*^					22–28 mm^*a*^	S^*a*^		
Piperacillin/tazobactam			1/4–4/4 mg/L	S					1/4–8/4 mg/L	S		
Meropenem			0.008–0.06 mg/L	S		S^*b*^	0.03–0.25 mg/L	S	0.12–1 mg/L	S		
Amikacin			0.5–4 mg/L	S					1–4 mg/L	S		
Vancomycin	1–4 mg/L	S			0.5–2 mg/L	S	0.12–0.5 mg/L	S				

^*a*^ The breakpoints of the cefoperazone/sulbactam disk diffusion method refer to the cefoperazone breakpoints (12) (resistant: ≤15 mm; intermediate: 16–20 mm; sensitive: ≥21 mm), and the quality control range refers to the literature (13).

^*b*^ *Staphylococcus aureus* ATCC29213 is a methicillin-susceptible *Staphylococcus aureus* that is susceptible to meropenem according to the CLSI standards (14).

#### Blood culture instruments and blood culture bottles

The blood culture instruments used in this study included the BacT/Alert 3D and BacT/Alert VIRTUO systems (bioMérieux, Lyon, France) and the Bactec FX system (BD Diagnostics, New Jersey, USA). The BacT/Alert-SA, BacT/Alert-PF, and BacT/Alert-PF plus bottles were obtained from bioMérieux, and the Bactec-PF bottles were obtained from BD.

#### Others

Sterile horse blood, blood agar, chocolate agar, and Sabouraud agar plates were purchased from Guangzhou Dijing Company. Ten antibiotics commonly used in pediatric clinical practice—ampicillin, oxacillin, ceftriaxone, ceftazidime, cefepime, cefoperazone/sulbactam, piperacillin/tazobactam, meropenem, amikacin, and vancomycin—were selected for testing. Antibiotic standards were obtained from the China National Institutes of Food and Drug Control, and the corresponding solvents for solution preparation were purchased from Shanghai Sinopharm Chemical Reagent Co., Ltd. (see Table 2).

**TABLE 2:** Antibiotic adsorption evaluation test: antibiotic peak plasma concentrations, solvents, and diluents

Antibiotic	Antibiotic concentration^*a*^ (mg/L)	Antibiotic solvent	Antibiotic diluent
Ampicillin	47	pH 8 0.1 mmol/L phosphate buffer	pH 6 0.1 mmol/L phosphate buffer
Oxacillin	43	Water	Water
Ceftriaxone	150	Water	Water
Ceftazidime	69	Sodium carbonate	Water
Cefepime	164	pH 6 0.1 mmol/L phosphate buffer	pH 6 0.1 mmol/L phosphate buffer
Cefoperazone/sulbactam	236.8/130.2	Water	Water
Piperacillin/tazobactam	242/24	Water	Water
Meropenem	49	Water	Water
Amikacin	30	Water	Water
Vancomycin	50	Water	Water

^*a*^ For the peak plasma concentration of ampicillin, see reference (15); for that of cefoperazone/sulbactam, see the reference package insert; and for other antibiotics, see reference (16).

### Method

#### Preparation of bacterial and fungal suspensions

Clinical isolates and ATCC reference strains were retrieved from –80°C storage and inoculated onto the appropriate agar media (blood agar, chocolate agar, or Sabouraud agar). After two consecutive overnight incubations at 35°C in ambient air or 5% CO₂, three to five colonies were suspended in 0.9% sodium chloride (NaCl) solution and adjusted to a turbidity equivalent to 0.5 McFarland standard (corresponding to ~10⁸ CFU/mL for bacteria and ~10⁶ CFU/mL for yeasts). The bacterial suspensions were serially diluted three times at 1:100, and the yeast suspensions were diluted twice at 1:100 to obtain a final concentration of approximately 10² CFU/mL.

#### TTD evaluation test

For TTD evaluation, 4 mL of sterile horse blood and 0.3 mL of each bacterial or fungal suspension were inoculated into four pediatric blood culture bottles as follows: one BacT/Alert-PF bottle, two BacT/Alert-PF plus bottles, and one Bactec-PF bottle. The final concentration of microorganisms in inoculated horse blood is approximately 7 CFU/mL, which is comparable to the bacterial loads typically observed in pediatric bloodstream infections (17, 18).

**Fig 1:**
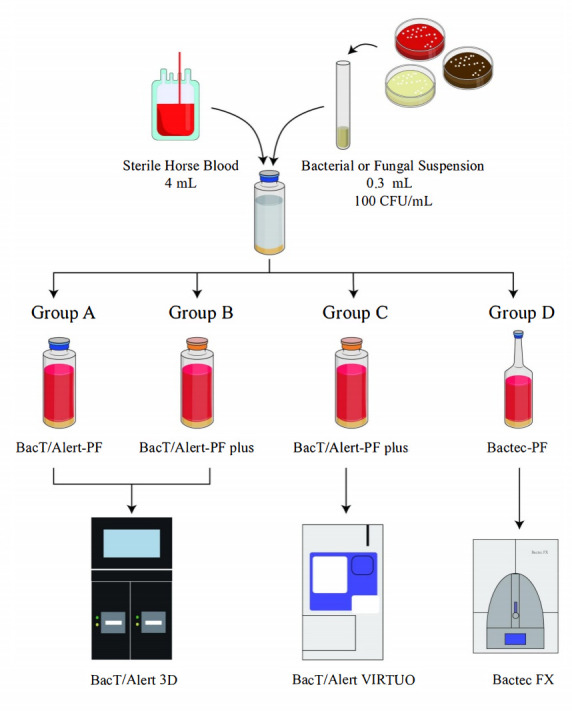
Time to detection evaluation test.

One BacT/Alert-PF bottle and one BacT/Alert-PF plus bottle were incubated in the BacT/Alert 3D instrument (designated groups A and B, respectively). The second BacT/Alert-PF plus bottle was incubated in the BacT/Alert VIRTUO instrument (group C), and the Bactec-PF bottle was incubated in the Bactec FX instrument (group D). The TTDs of all four bottles inoculated with each strain were recorded (see Fig. 1).

#### Antibiotic adsorption evaluation test

A total of 23 antibiotic–ATCC strain combinations, each comprising an ATCC reference strain susceptible to the corresponding antibiotic, were evaluated (see Table 1). Based on previous reports and the availability of well-established MIC ranges for ATCC strains, clinical isolates were not included in this evaluation (9, 10, 19). Antibiotic standards were dissolved and diluted using the corresponding solvents specified in the Clinical and Laboratory Standards Institute (CLSI) standards (14). The final antibiotic concentrations were prepared at eight times the reported peak plasma concentration, as referenced in the literature or in the respective drug package inserts (15, 16) (see Table 2).

**Fig 2:**
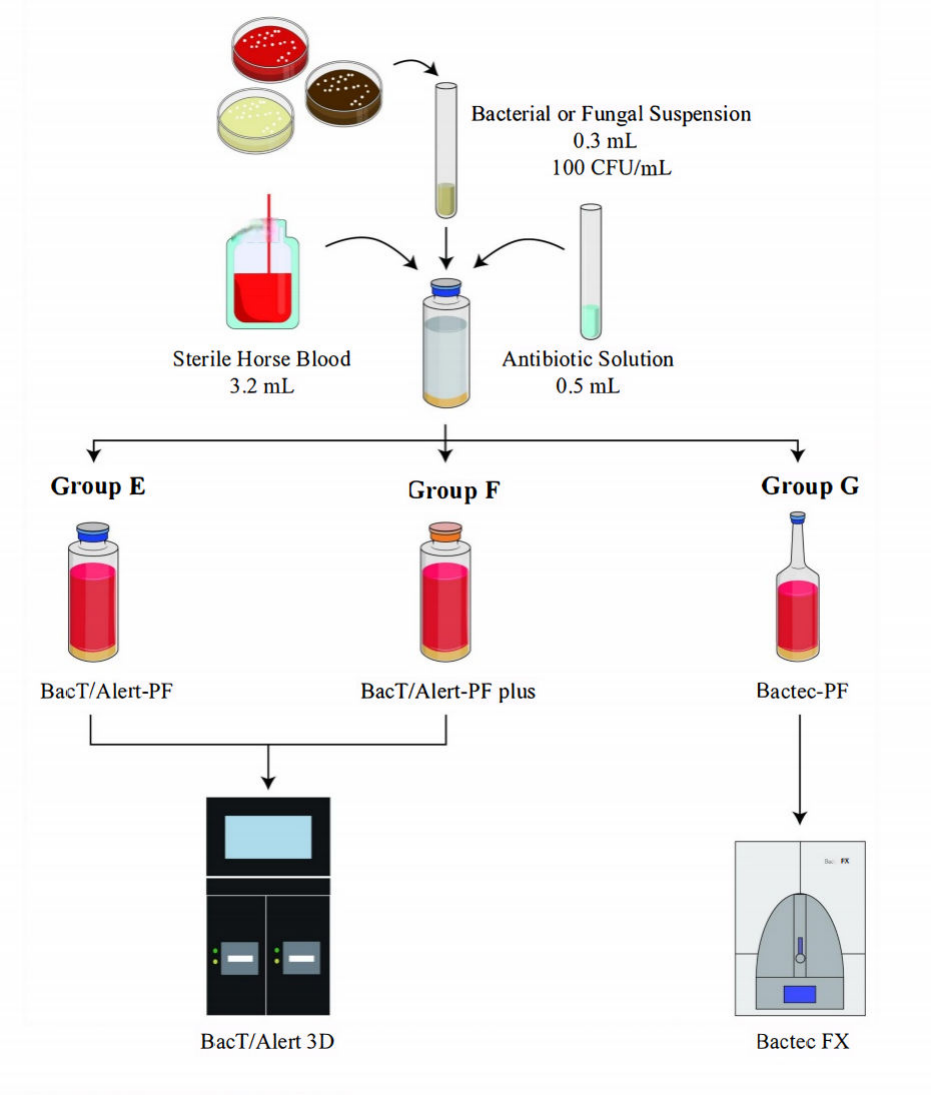
Antibiotic adsorption evaluation test.

For inoculation, 3.2 mL of sterile horse blood, 0.3 mL of the prepared bacterial or fungal suspension, and 0.5 mL of antibiotic solution were added sequentially into 15 blood culture bottles (five each of BacT/Alert-PF, BacT/Alert-PF plus, and Bactec-PF). The final microbial concentration in horse blood was approximately 8 CFU/mL, while the antibiotic concentrations corresponded to peak plasma levels after an eightfold dilution. BacT/Alert-PF and BacT/Alert-PF plus bottles were incubated in the BacT/Alert 3D instrument (designated groups E and F, respectively), and Bactec-PF bottles were incubated in the Bactec FX instrument (group G) (see Fig. 2). The cultures were incubated for up to five days or until flagged positive, and the results were recorded.

#### Quality control

For quality control, 0.3 mL of the diluted bacterial or fungal suspension was inoculated onto the corresponding culture medium for colony counting. Plates yielding 5–100 CFU confirmed that the bacterial suspension dilutions were appropriately controlled. Each batch of TTD evaluation samples included a sterile control bottle that was inoculated sequentially with 4 mL of sterile horse blood and 0.3 mL of 0.9% NaCl solution.

For the antibiotic adsorption evaluation, three types of quality control bottles were included for each bacteria–antibiotic combination: (i) a sterile control bottle containing 3.2 mL of sterile horse blood and 0.5 mL of antibiotic solution; (ii) a positive control bottle containing 3.2 mL of sterile horse blood and 0.3 mL of the prepared bacterial or fungal suspension; and (iii) a negative control (BacT/Alert-SA standard bottle, bioMérieux) containing 3.2 mL of sterile horse blood, 0.3 mL of bacterial or fungal suspension, and 0.5 mL of antibiotic solution, which was used to confirm antibiotic effectiveness. Positive bottles from the tests were subcultured onto the corresponding agar plates to verify strain identification and purity.

#### Data statistical analysis

For the TTD evaluation, the median and interquartile range (IQR) of TTDs were calculated for each strain (species or spp.) across the four detection schemes. TTDs between groups were compared via the rank-sum test.

For the antibiotic adsorption evaluation, the positive detection rates and TTDs of the three pediatric blood culture bottles were calculated for each antibiotic–ATCC strain combination. Positive detection rates between groups were compared via Fisher’s exact test. A *P* value ≤ 0.05 was considered statistically significant.

## RESULTS

### TTD evaluation tests

Among all the tested bacteria, BacT/Alert-PF plus incubation in the BacT/Alert 3D system shortened the overall median TTD by 3.33 h (13.92 vs 17.25, *P* < 0.0001) compared with BacT/Alert-PF. Significant reductions were observed for *Enterococcus* spp., *Enterobacteriaceae*, nonfermenting gram-negative bacilli, and rare/fastidious bacteria. For example, the median TTD of *Acinetobacter baumannii* was reduced by 15.43 h (11.96 vs 27.39, *P* = 0.006).

Compared with the BacT/Alert 3D system, the BacT/Alert VIRTUO system with BacT/Alert-PF plus (group C vs group B) shortened the overall median TTD by 2.3 h across all tested bacteria (11.62 vs. 13.92 h, *P* < 0.0001). Significant differences were observed for *Staphylococcus* spp. (13.31 vs 16.02 h, *P* = 0.0034), *Streptococcus* spp. (9.79 vs 11.88 h, *P* = 0.0013), *Enterococcus* spp. (12.32 vs14.84 h, *P* = 0.0016), and *Enterobacteriaceae* (9.38 vs 12.33 h, *P* < 0.0001). At the species level, the median TTDs of *Staphylococcus aureus*, *Streptococcus pyogenes*, *Streptococcus agalactiae*, *Streptococcus pneumoniae*, and *Enterococcus faecium* were shortened by 2.34 h (*P* = 0.015), 1.92 h (*P* = 0.0445), 2.42 h (*P* = 0.0025), 2.8 h (*P* = 0.0109), and 2.78 h (*P* = 0.0064), respectively. Among gram-negative bacilli, *Escherichia coli*, *Klebsiella pneumoniae*, *Enterobacter cloacae* complex, *Salmonella* spp., and *Pseudomonas aeruginosa* were reduced by 2.9 h (*P* = 0.0008), 2.33 h (*P* = 0.0019), 2.49 h (*P* = 0.0327), 2.3 h (*P* = 0.0019), and 2.77 h (*P* = 0.0032), respectively.

When BacT/Alert VIRTUO with BacT/Alert-PF plus was compared with BacT/Alert 3D with BacT/Alert-PF (group C vs. group A), the overall median TTD was shortened by 5.63 h (11.62 vs 17.25 h, *P* < 0.0001). The median TTDs were similar only for *Streptococcus constellatus/anginosus* (21.85 vs 22.10 h, *P* = 1.0) and *Stenotrophomonas maltophilia* (19.50 vs 20.83 h, *P* = 1.0).

Compared with the Bactec FX system with Bactec-PF (group D), BacT/Alert VIRTUO with BacT/Alert-PF plus (group C) shortened the overall median TTD by 1.86 h (11.62 vs 13.48 h, *P* = 0.0014). The greatest reductions were observed for nonfermenting gram-negative bacilli (3.8 h; 13.93 vs 17.73 h, *P* = 0.0031) and *A. baumannii* (11.69 h; 9.47 vs 21.16 h, *P* = 0.0007).

The overall TTD of *Candida* spp. did not show consistent or clinically meaningful differences across the four groups. However, several species-specific comparisons revealed statistically significant differences. For example, when BacT/Alert VIRTUO with BacT/Alert-PF plus was compared with BacT/Alert 3D with BacT/Alert-PF (group C vs group A), the median TTD was reduced by 5.67 h for *Candida glabrata* (14.85 vs 20.52 h, *P* = 0.045) and by 1.88 h for *Candida tropicalis* (15.30 vs 17.18 h, *P* = 0.0196). Similarly, compared with Bactec FX with Bactec-PF (group C vs group D), the median TTD for *C. glabrata* was shortened by 40.68 h (14.85 vs 55.53 h, *P* = 0.0004), whereas that for *C. parapsilosis* was prolonged by 3.47 h (33.25 vs 29.78 h, *P* = 0.0073) (Table 3). Taken together, these findings suggest that although certain species-specific differences were statistically significant, the patterns were inconsistent across *Candida* species.

**TABLE 3:** Differences in the TTD of blood cultures of various strains in the three blood culture systems and three blood culture bottles^*b*^

Organism	No.	TTD (hours), median (interquartile range)				*P* value^*a*^			
		Group A	Group B	Group C	Group D	Group A vs B	Group B vs C	Group A vs C	Group C vs D
		BacT/Alert 3D		BacT/Alert VIRTUO	Bactec FX				
		BacT/Alert–PF	BacT/Alert–PF plus		Bactec–PF				
All bacteria	183	17.25 (13.82–20.88)	13.92 (12.15–17.52)	11.62 (9.78–15)	13.48 (10.68–18.16)	**<0.0001**	**<0.0001**	**<0.0001**	**0.0014**
*Staphylococcus* spp.	20	17.31 (14.98–20.75)	16.02 (14.35–17.32)	13.31 (11.88–14.10)	14.88 (12.84–16.77)	1	**0.0034**	**<0.0001**	0.2597
*Staphylococcus aureus*	10	15.00 (14.46–16.21)	14.43 (13.33–15.96)	12.09 (11.00–13.12)	13.04 (11.61–13.84)	1	**0.0150**	**0.0004**	1
Coagulase-negative *staphylococci*	10	20.20 (17.79–24.60)	17.29 (16.24–18.51)	13.90 (13.32–15.02)	16.54 (15.03–18.46)	0.8448	0.0589	**0.0003**	0.2676
*Streptococcus* spp.	38	14.11 (12.12–16.69)	11.88 (10.91–13.99)	9.79 (8.80–11.16)	10.94 (9.40–12.99)	0.2095	**0.0013**	**<0.0001**	1
*Streptococcus pyogenes*	5	13.88 (13.38–14.64)	11.55 (11.38–11.88)	9.63 (9.63–9.72)	10.40 (10.16–10.80)	1	**0.0445**	**0.0004**	1
*Streptococcus agalactiae*	10	11.86 (11.04–13.02)	10.26 (10.11–10.33)	7.84 (7.54–8.14)	8.68 (8.42–9.01)	0.3341	**0.0025**	**<0.0001**	0.3491
*Streptococcus constellatus/anginosus*	5	22.10 (21.16–24.08)	22.40 (17.34–31.42)	21.85 (14.87–36.38)	19.58 (14.99–22.05)	1	1	1	1
*Streptococcus dysgalactiae*	5	11.97 (11.55–12.09)	11.42 (11.20–11.62)	9.77 (9.37–10.43)	9.83 (9.53–10.15)	1	0.1121	**0.0067**	1
*Streptococcus pneumoniae*	10	14.76 (14.29–16.12)	13.73 (12.95–14.30)	10.93 (10.37–11.16)	11.51 (11.03–12.16)	1	**0.0109**	**<0.0001**	1
*Streptococcus mitis/oral*	3	17.52 (16.56–21.73)	13.90 (13.68–15.84)	12.02 (10.73–13.08)	14.97 (13.62–15.62)	0.8461	0.6775	**0.0134**	0.8461
*Enterococcus* spp.	20	19.84 (17.32–20.99)	14.84 (13.59–15.69)	12.32 (10.87–13.05)	12.99 (11.73–13.67)	**0.0120**	**0.0016**	**<0.0001**	1
*Enterococcus faecalis*	10	18.90 (16.94–20.76)	14.34 (13.42–16.38)	12.45 (10.66–14.36)	12.20 (11.40–13.30)	0.1669	0.3202	**0.0002**	1
*Enterococcus faecium*	10	20.25 (18.08–21.16)	15.10 (14.05–15.48)	12.32 (11.26–12.53)	13.54 (12.84–13.71)	0.1929	**0.0064**	**<0.0001**	0.7005
*Enterobacteriaceae*	45	13.33 (12.55–14.60)	12.33 (11.46–12.69)	9.38 (8.80–10.48)	10.23 (9.63–11.29)	**0.0155**	**<0.0001**	**<0.0001**	0.3806
*Escherichia coli*	10	13.15 (12.73–13.60)	11.59 (11.38–12.02)	8.69 (8.45–9.22)	9.69 (9.42–10.27)	0.3979	**0.0008**	**<0.0001**	0.3979
*Klebsiella pneumoniae*	10	12.43 (12.20–13.02)	11.26 (11.04–12.02)	8.93 (8.67–9.52)	9.74 (9.58–10.15)	0.6481	**0.0019**	**<0.0001**	1
*Enterobacter cloacae* complex	5	13.22 (12.39–13.93)	12.52 (11.96–13.18)	10.03 (9.54–10.96)	11.28 (10.09–11.58)	1	**0.0327**	**0.0037**	1
*Klebsiella aerogenes*	5	12.63 (12.48–13.72)	12.32 (11.90–12.47)	10.13 (8.99–10.16)	10.33 (9.97–10.50)	1	0.0611	**0.0009**	1
*Klebsiella oxytoca*	5	13.82 (13.27–15.95)	12.50 (11.76–13.94)	9.83 (9.03–11.47)	10.13 (7.87–11.66)	1	0.3259	**0.0139**	1
*Salmonella* spp.	10	15.56 (15.00–16.25)	12.86 (12.64–13.30)	10.56 (10.45–11.05)	11.34 (10.88–11.51)	0.4325	**0.0019**	**<0.0001**	1
Nonfermenting gram-negative bacilli	25	20.72 (17.68–26.82)	16.93 (12.18–19.17)	13.93 (9.65–15.48)	17.73 (15.31–21.07)	**0.0043**	0.4277	**<0.0001**	**0.0031**
*Pseudomonas aeruginosa*	10	17.86 (17.44–18.56)	17.59 (16.95–18.81)	14.82 (14.19–15.38)	15.40 (15.26–16.11)	1	**0.0032**	**0.0005**	1
*Acinetobacter baumannii*	10	27.39 (24.18–34.82)	11.96 (11.63–12.57)	9.47 (9.03–10.64)	21.16 (20.66–25.86)	**0.0060**	0.2674	**<0.0001**	**0.0007**
*Stenotrophomonas maltophilia*	5	20.83 (17.45–21.14)	21.85 (16.53–25.61)	19.50 (13.82–23.99)	18.38 (15.19–20.58)	1	1	1	1
Rare and fastidious bacteria	10	20.09 (19.85–21.93)	17.87 (16.92–18.28)	15.05 (14.10–16.36)	18.16 (16.19–21.01)	**0.0254**	0.3420	**<0.0001**	**0.0457**
*Haemophilus influenzae*	5	21.32 (20.12–26.64)	17.52 (14.52–18.48)	15.02 (12.70–17.02)	18.22 (14.76–19.44)	0.0836	1	**0.0046**	0.9876
*Moraxella catarrhalis*	5	19.92 (19.39–20.09)	18.07 (17.63–18.44)	15.08 (14.49–16.10)	18.10 (16.78–23.88)	0.8069	0.2878	**0.0031**	0.0618
*Candida* spp.	25	24.35 (19.70–29.82)	23.68 (16.86–29.32)	23.30 (15.23–29.31)	28.77 (22.94–32.50)	1	1	1	0.1459
*Candida albicans*	10	27.09 (24.31–28.62)	25.82 (23.67–27.69)	25.70 (23.01–27.29)	28.07 (23.74–29.18)	1	1	1	1
*Candida glabrata*	5	20.52 (19.70–21.02)	16.85 (16.69–17.44)	14.85 (13.81–15.39)	55.53 (54.81–57.34)	1	1	**0.0450**	**0.0004**
*Candida parapsilosis*	5	31.33 (31.03–32.98)	31.17 (30.28–32.57)	33.25 (32.02–34.09)	29.78 (29.17–31.07)	1	0.3676	1	**0.0073**
*Candida tropicalis*	5	17.18 (16.91–17.97)	16.67 (15.40–17.60)	15.30 (14.20–15.63)	14.63 (14.62–15.85)	1	0.4658	**0.0196**	1

^*a*^ The TTD among each group was compared using the rank sum test. A *P* value ≤ 0.05 was considered statistically significant, and the bold test indicates that the corresponding *P*-values are statistically significant.

^*b*^ Including five strains of *Staphylococcus epidermidis*, two strains of *Staphylococcus hominis*, two strains of *Staphylococcus hemolyticus*, and one strain of *Staphylococcus capitis*.

### Antibiotic adsorption evaluation

At the peak plasma concentration of antibiotics, the overall detection rate of BacT/Alert-PF (13%, 15/115) was significantly lower than that of BacT/Alert-PF plus (46.1%, 53/115) or Bactec-PF (51.3%, 59/115), both *P* < 0.0001. The detection rate of BacT/Alert-PF plus was similar to that of Bactec-PF (46.1% vs 51.3%, *P* = 0.5096).

For gram-positive cocci, the detection rates with BacT/Alert-PF, BacT/Alert-PF plus, and Bactec-PF were 12.5% (5/40), 57.5% (23/40), and 45.0% (18/40), respectively. For gram-negative bacilli, the rates were 13.3% (10/75), 40.0% (30/75), and 54.7% (41/75), respectively.

In the ceftriaxone and meropenem groups, all three bottles (BacT/Alert-PF, BacT/Alert-PF plus, and Bactec-PF) presented a detection rate of 0%. In contrast, in the amikacin group, all bottles achieved a detection rate of 100%. Among the other antibiotic groups, BacT/Alert-PF demonstrated very low recoveries, with recoveries of 33.3% in the vancomycin group and 0% in all the other groups.

Compared with Bactec-PF, BacT/Alert-PF plus exhibited higher detection rates for ampicillin, piperacillin/tazobactam, and vancomycin. In contrast, the detection rates in the ceftazidime, cefepime, and cefoperazone/sulbactam groups were uniformly low. For oxacillin, Bactec-PF and BacT/Alert-PF plus bottles achieved a detection rate of 100%.

In addition, the results indicated that increasing antimicrobial resistance was one of the factors influencing detection rates. For example, in the ampicillin group, the detection rate of Bactec-PF increased from 20% for *Enterococcus faecalis* ATCC 29212 (MIC: 0.5–2 mg/L) to 100% for *Escherichia coli* ATCC 25922 (MIC: 2–8 mg/L). In the ceftazidime group, the detection rate of Bactec-PF increased from 20% for *Escherichia coli* ATCC 25922 (MIC: 0.06–0.5 mg/L) to 100% for *Pseudomonas aeruginosa* ATCC 27853 (MIC: 1–4 mg/L). In the cefoperazone/sulbactam group, the detection rates of BacT/Alert-PF plus and Bactec-PF increased from 0% and 40%, respectively, for *Escherichia coli* ATCC25922 (inhibition zone diameter: 27–33 mm) to 100% and 100%, respectively, for *Pseudomonas aeruginosa* ATCC27853 (inhibition zone diameter: 22–28 mm). In the vancomycin group, the detection rates of BacT/Alert-PF plus and Bactec-PF increased from 60% and 40%, respectively, for *Streptococcus pneumoniae* ATCC 49619 (MIC: 0.12–0.5 mg/L) to 100% and 100%, respectively, for *Staphylococcus aureus* ATCC29213 (MIC: 0.5–2 mg/L).

In addition, the results showed that BacT/Alert-PF plus and Bactec-PF resulted in differences in TTDs under certain antibiotic–bacteria combinations. Compared with BacT/Alert-PF plus, Bactec-PF prolonged the median TTD of *Staphylococcus aureus* ATCC 29213 by 7.8 h (22.17 vs 14.37) and 9.65 h (23.85 vs 14.20) in the oxacillin and vancomycin groups, respectively. In the vancomycin group, the median TTD of *Streptococcus pneumoniae* ATCC 49619 was also increased by 9.15 h compared with that of the BacT/Alert-PF plus group (30.97 vs 21.82). For *Pseudomonas aeruginosa* ATCC 27853, the median TTD was markedly prolonged in the ceftazidime and cefepime groups, by 15.7 h (32.58 vs 16.88) and 23.39 h (40.27 vs 16.88), respectively. In contrast, little difference in TTD was observed between Bactec-PF and BacT/Alert-PF plus in the other test groups (Table 4).

**TABLE 4:** Differences in the adsorption performance of BacT/Alert-PF, BacT/Alert-PF plus, and Bactec-PF for various antibiotics

Antibiotic	ATCC strains	MIC range/ Inhibition zone diameter	Detection rate (%)			*P* value of the detection rate^*a*^			TTD (hours), median (interquartile range)		
			Group E	Group F	Group G				Group E	Group F	Group G
			BacT/Alert-PF	BacT/Alert-PF plus	Bactec-PF	Group E vs F	Group E vs G	Group F vs G	BacT/Alert-PF	BacT/Alert-PF plus	Bactec-PF
Ampicillin	*Enterococcus faecalis* ATCC29212	0.5–2 mg/L	0/5 (0)	5/5 (100)	1/5 (20)	**0.0079**	1	**0.0476**	–^*b*^	14.22 (13.88–14.38)	18.27
	*Escherichia coli* ATCC25922	2–8 mg/L	0/5 (0)	5/5 (100)	5/5 (100)	**0.0079**	**0.0079**	1	–	12.58 (12.35–12.59)	11.28 (10.89–11.32)
Oxacillin	*Staphylococcus aureus* ATCC29213	0.12–0.5 mg/L	0/5 (0)	5/5 (100)	5/5 (100)	**0.0079**	**0.0079**	1	–	14.37 (14.12–14.54)	22.17 (20.42–22.91)
Ceftriaxone	*Escherichia coli* ATCC25922	0.03–0.12 mg/L	0/5 (0)	0/5 (0)	0/5 (0)	1	1	1	–	–	–
	*Streptococcus pneumoniae* ATCC49619	0.03–0.12 mg/L	0/5 (0)	0/5 (0)	0/5 (0)	1	1	1	–	–	–
Ceftazidime	*Escherichia coli* ATCC25922	0.06–0.5 mg/L	0/5 (0)	0/5 (0)	1/5 (20)	1	1	1	–	–	11.88
	*Pseudomonas aeruginosa* ATCC27853	1–4 mg/L	0/5 (0)	0/5 (0)	5/5 (100)	1	**0.0079**	**0.0079**	–	–	32.58 (19.39–40.69)
Cefepime	*Escherichia coli* ATCC25922	0.016–0.12 mg/L	0/5 (0)	0/5 (0)	0/5 (0)	1	1	1	–	–	–
	*Klebsiella pneumoniae* ATCC700603	0.5–2 mg/L	0/5 (0)	0/5 (0)	0/5 (0)	1	1	1	–	–	–
	*Pseudomonas aeruginosa* ATCC27853	0.5–4 mg/L	0/5 (0)	0/5 (0)	5/5 (100)	1	**0.0079**	**0.0079**	–	–	40.27 (22.94–45.42)
Cefoperazone/sulbactam	*Escherichia coli* ATCC25922	27–33 mm	0/5 (0)	0/5 (0)	2/5 (40)	1	0.444	0.444	–	–	13.89 (13.22–14.55)
	*Pseudomonas aeruginosa* ATCC27853	22–28 mm	0/5 (0)	5/5 (100)	5/5 (100)	**0.0079**	**0.0079**	1	–	14.50 (14.34–14.64)	14.97 (14.63–15.12)
Piperacillin/tazobactam	*Escherichia coli* ATCC25922	1/4–4/4 mg/L	0/5 (0)	5/5 (100)	3/5 (60)	**0.0079**	0.167	0.444	–	11.83 (11.58–11.95)	12.22 (11.72–12.73)
	*Pseudomonas aeruginosa* ATCC27853	1/4–8/4 mg/L	0/5 (0)	5/5 (100)	5/5 (100)	**0.0079**	**0.0079**	1	–	15.88 (15.86–16.44)	16.88 (16.05–17.15)
Meropenem	*Staphylococcus aureus* ATCC29213	–	0/5 (0)	0/5 (0)	0/5 (0)	1	1	1	–	–	–
	*Escherichia coli* ATCC25922	0.008–0.06 mg/L	0/5 (0)	0/5 (0)	0/5 (0)	1	1	1	–	–	–
	*Streptococcus pneumoniae* ATCC49619	0.03–0.25 mg/L	0/5 (0)	0/5 (0)	0/5 (0)	1	1	1	–	–	–
	*Pseudomonas aeruginosa* ATCC27853	0.12–1 mg/L	0/5 (0)	0/5 (0)	0/5 (0)	1	1	1	–	–	–
Amikacin	*Escherichia coli* ATCC25922	0.5–4 mg/L	5/5 (100)	5/5 (100)	5/5 (100)	1	1	1	13.68 (13.36–13.76)	12.53 (12.17–12.71)	10.78 (10.69–10.94)
	*Pseudomonas aeruginosa* ATCC27853	1–4 mg/L	5/5 (100)	5/5 (100)	5/5 (100)	1	1	1	17.08 (16.82–17.23)	16.05 (15.97–16.21)	15.60 (15.58–15.85)
Vancomycin	*Streptococcus pneumoniae* ATCC49619	0.12–0.5 mg/L	0/5 (0)	3/5 (60)	2/5 (40)	0.167	0.444	1	–	21.82 (18.33–31.67)	30.97 (29.85–32.08)
	*Staphylococcus aureus* ATCC29213	0.5–2 mg/L	0/5 (0)	5/5 (100)	5/5 (100)	**0.0079**	**0.0079**	1	–	14.20 (14.10–14.62)	23.85 (21.13–25.96)
	*Enterococcus faecalis* ATCC29212	1–4 mg/L	5/5 (100)	5/5 (100)	5/5 (100)	1	1	1	44.83 (40.49–48.92)	13.50 (13.33–13.50)	12.02 (11.59–12.16)

^*a*^ The positive rates among each group were compared using Fisher’s exact test. A *P* value ≤ 0.05 was considered statistically significant, and the bold test indicates that the corresponding *P*-values are statistically significant.

^*b*^ –, not applicable.

## DISCUSSION

Bloodstream infections constitute one of the major causes of the global disease burden, as severe systemic infectious diseases are prone to induce sepsis (20). There are an estimated 575,000–677,000 episodes of bloodstream infection (BSI) per year in North America and 79,000–94,000 deaths and over 1,200,000 episodes of BSI and 157,000 deaths per year in Europe (21). Timely and effective antimicrobial therapy has been shown to significantly reduce mortality in BSI patients (22).

However, selecting appropriate antibiotics before etiological results are available (including pathogen identification and antimicrobial susceptibility testing) remains a major clinical challenge. To ensure coverage of possible pathogens and multidrug-resistant organisms, clinicians often initiate empiric therapy with broad-spectrum antibiotics. While effective, this strategy is a double-edged sword owing to potential toxic side effects, disruption of normal flora, and the induction of resistance to broad-spectrum agents. Rapid etiological reporting enables the transition from empiric to targeted therapy—shifting from combination to monotherapy and from broad-spectrum to narrow-spectrum antibiotics. This approach not only minimizes the toxic side effects associated with broad-spectrum antibiotics but also reduces the economic burden on patients.

Our findings demonstrate that the BacT/Alert VIRTUO system with BacT/Alert-PF plus bottles significantly reduced the TTD compared with the BacT/Alert 3D system using BacT/Alert-PF bottles. Under identical BacT/Alert 3D conditions, BacT/Alert-PF plus bottles also yielded shorter median TTDs than BacT/Alert-PF bottles did, although the reduction (3.33 h) was slightly smaller than the 4.9 h reported in previous studies (23). Notably, TTD reductions were consistent across most bacterial groups, except for *Streptococcus constellatus/anginosus* and *Stenotrophomonas maltophilia*. The limited improvement in *S. constellatus/anginosus* may reflect its inherently slower growth rate. Moreover, a previous study comparing VIRTUO and 3D under identical bottle conditions reported reduced median TTDs for all microorganisms except *S. maltophilia*, for which VIRTUO presented a 7.2 h longer TTD (3). These findings suggest that further optimization of blood culture bottles or detection algorithms may be needed for this species. Under identical BacT/Alert-PF plus bottle conditions, VIRTUO shortened the overall bacterial median TTD by 2.3 h compared with 3D, which is consistent with previous reports (3).

When comparing BacT/Alert VIRTUO with BacT/Alert-PF plus to BD Bactec FX with Bactec-PF, the overall bacterial median TTD was shortened by 1.86 h, similar to literature reports (4, 24). The median TTDs were reduced for most test groups except *S. constellatus/anginosus*, *Enterococcus faecalis*, *Stenotrophomonas maltophilia*, *Candida parapsilosis*, and *Candida tropicalis*. Notably, the median TTDs for *Acinetobacter baumannii* and *Candida glabrata* were shortened by 11.69 h and 40.68 h, respectively. The pronounced decrease in TTD for *C. glabrata* may be related to the 2018 reformulation of the BacT/Alert-PF plus bottle, which reportedly shortened its average TTD by approximately 30 h (8). Although some individual *Candida* species showed statistically significant differences in TTD, these differences were not consistent across all *Candida* spp. Taken together, the overall TTD for *Candida* spp. as a group did not differ significantly, which is consistent with previous reports (3, 25).

The 2018 updated Infectious Diseases Society of America (IDSA) and American Society for Microbiology Infectious Disease Diagnostic Guidelines for Clinical Microbiology Laboratories (26) and the 2021 Surviving Sepsis Campaign International Sepsis and Septic Shock Guidelines (27) recommend collecting blood cultures and sending them for testing before antibiotic treatment. However, in clinical practice, fully achieving this goal is often challenging. The collection of blood cultures after antibiotic use is a major contributor to reduced positivity rates.

In our antibiotic adsorption evaluation, the detection rate of BacT/Alert-PF was significantly lower than that of either BacT/Alert-PF plus or Bactec-PF, which is consistent with previous reports (28). The overall detection rate of BacT/Alert-PF plus is slightly lower than that of Bactec-PF, which is different from a previous literature report demonstrating that the detection rate of BacT/Alert-PF plus is higher than that of Bactec-PF (54.7% vs 36%) (9). However, the detection rates of piperacillin/tazobactam-*Pseudomonas aeruginosa* ATCC27853 and oxacillin-*Staphylococcus aureus* ATCC29213 were both 100%, whereas those of ceftriaxone-*Escherichia coli* ATCC25922, *Streptococcus pneumoniae* ATCC49619, and cefepime-*Escherichia coli* ATCC25922 were 0%, similar to those reported in the literature (9).

Several studies have compared the detection rates of adult blood culture bottles (BacT/Alert-FA plus and Bactec-FA) in specific antibiotic–bacteria combinations, including ampicillin-*Enterococcus faecalis* ATCC 29212, cefepime-*Escherichia coli* ATCC 25922 (19), ceftriaxone-*Escherichia coli* ATCC 25922 (19), oxacillin-*Staphylococcus aureus* ATCC 29213 (19), vancomycin-*Staphylococcus aureus* ATCC 29213 (19), *Enterococcus faecalis* ATCC 29212 (19, 29), piperacillin/tazobactam-*Pseudomonas aeruginosa* ATCC 27853 and *Escherichia coli* ATCC 25922 (29), and meropenem-*Pseudomonas aeruginosa* ATCC 27852 and *Escherichia coli* ATCC 25922 (29). The detection rates observed in these studies were largely consistent with those obtained in our study using pediatric bottles (BacT/Alert-PF plus and Bactec-PF) for the same combinations. However, in our study, the detection rate of Bactec-PF in the cefepime-*Pseudomonas aeruginosa* ATCC27853 and vancomycin-*Staphylococcus aureus* ATCC29213 combinations was 100%, which was higher than the 0% (19) and 40% (29) rates of Bactec-FA reported in the literature, respectively. Conversely, the detection rate of both BacT/Alert-PF plus and Bactec-PF for the meropenem-*Staphylococcus aureus* ATCC 29213 combination was 0%, which was lower than the 40% detection rate of BacT/Alert-FA plus and Bactec-FA reported in the literature (29).

In the amikacin group, all the tested ATCC strains were detected successfully in three blood culture bottles. This may be attributed primarily to the presence of the anticoagulant sodium polyanethole sulfonate (SPS) in the bottles, which can inactivate aminoglycoside antibiotics (30). Ceftriaxone, owing to its long half-life, requires infrequent dosing, whereas meropenem exhibits strong antibacterial activity against gram-negative bacilli and has fewer central nervous system adverse effects than does imipenem (31). Both antibiotics are commonly used in pediatric clinical practice. However, in the ceftriaxone and meropenem groups, none of the tested ATCC strains were detected in the three aerobic blood culture bottles. Previous studies have reported that BacT/Alert-FN plus anaerobic bottles have a stronger adsorption capacity for carbapenem antibiotics than aerobic bottles do (29), indicating that for children receiving carbapenem therapy, BacT/Alert-FN plus anaerobic bottles may be more suitable.

Moreover, in a variety of antibiotic test groups, even among different bacterial species, the detection rates of BacT/Alert-PF plus or Bactec-PF increased with increasing resistance of the ATCC strains to the corresponding antibiotics. This suggests that the degree of bacterial resistance to the corresponding antibiotics may have a more significant effect on the detection rate after the use of antibiotics than the differences in bacterial species do. The degree of bacterial resistance in blood cultures to the antibiotics used is also one of the main factors affecting the detection rate of antibiotics in blood cultures after antibiotic use.

Furthermore, across multiple antibiotic test groups and bacterial species, the detection rate of BacT/Alert-PF plus or Bactec-PF increased with the level of resistance of the ATCC strains to the corresponding antibiotics. These findings suggest that the susceptibility of bacteria in blood cultures to antibiotics is an important determinant of detection success following antibiotic exposure.

### Limitations

Because anaerobic bloodstream infections are uncommon in pediatric settings (18) and blood collection volumes in children are generally limited, it is difficult to routinely use paired aerobic and anaerobic bottles. Therefore, only aerobic pediatric bottles were evaluated in this *in vitro* study. However, some data show that the TTD of *S. aureus* in anaerobic bottles is shorter than that in aerobic bottles (32), which requires further research. In addition, sterile horse blood was used for simulation; however, given the physiological differences between equine and human blood, factors such as plasma protein binding may affect antibiotic activity. Thus, further validation in clinical pediatric settings is needed.

### Conclusions

In summary, this study compared three blood culture systems and three pediatric aerobic blood culture bottles. Both BacT/Alert VIRTUO with BacT/Alert-PF plus and Bactec FX with Bactec-PF showed shorter TTDs than BacT/Alert 3D with BacT/Alert-PF. In terms of antibiotic adsorption, BacT/Alert-PF plus and Bactec-PF demonstrated stronger adsorption than BacT/Alert-PF did, with comparable overall detection rates, although each system had specific strengths depending on the antibiotic–strain combination. These findings highlight the importance of selecting advanced culture systems and optimized bottles to improve blood culture yield and accelerate appropriate antimicrobial therapy in pediatric patients.
